# Supplementary figures and images for: Lyophilized cell-free supernatants of Lactobacillus isolates exhibited antibiofilm, antioxidant, and reduces nitric oxide activity in lipopolysaccharide-stimulated RAW 264.7 cells
Source: PeerJ. 2021 Nov 30;9:e12586. doi: 10.7717/peerj.12586 (PMC8641486; doi:10.7717/peerj.12586)

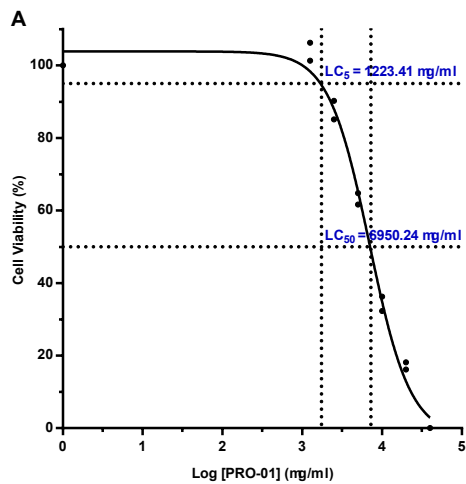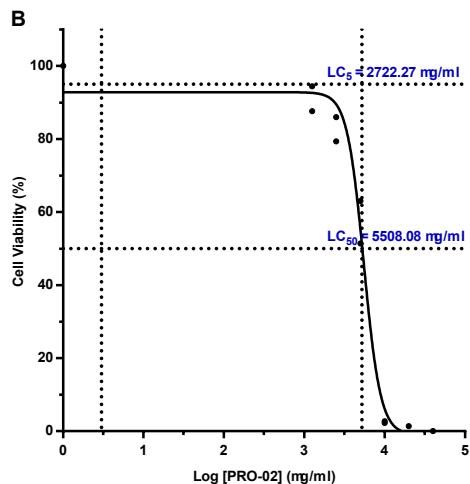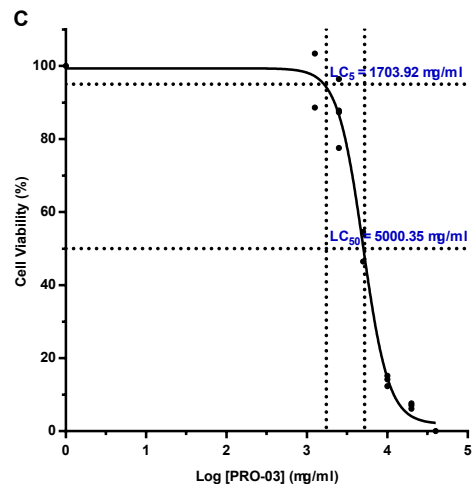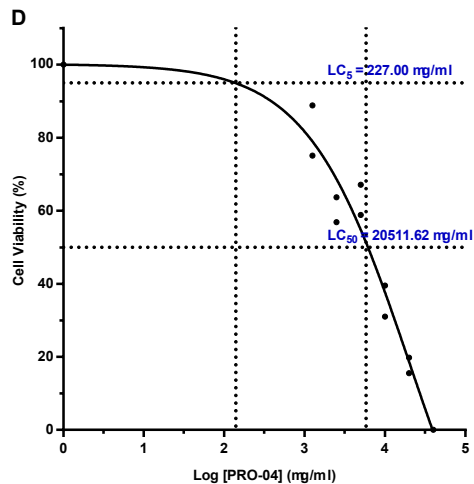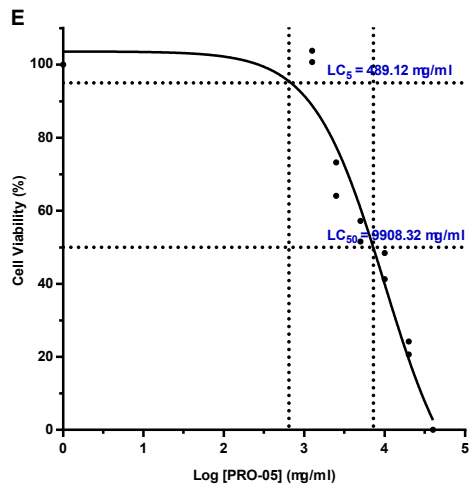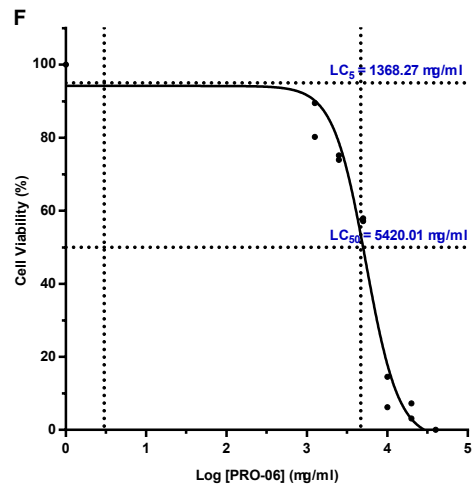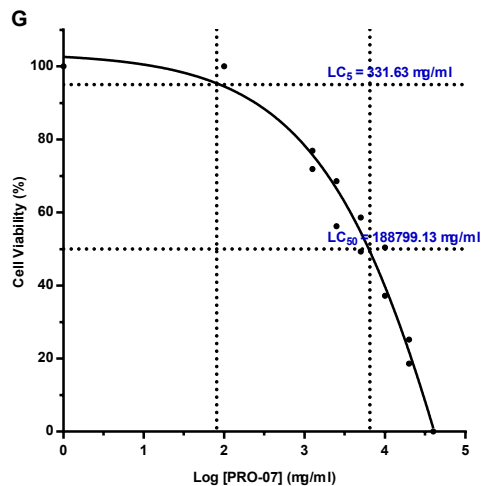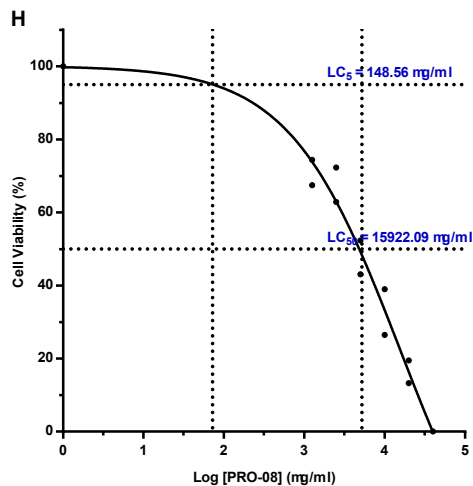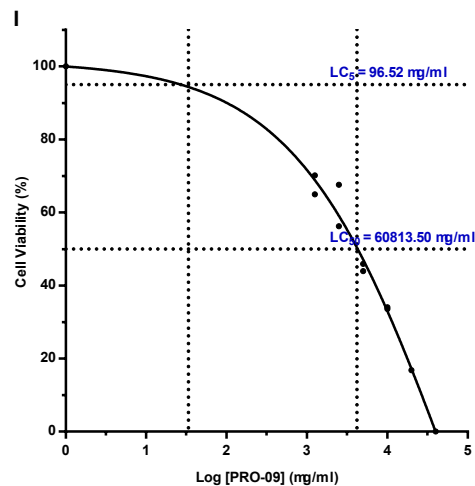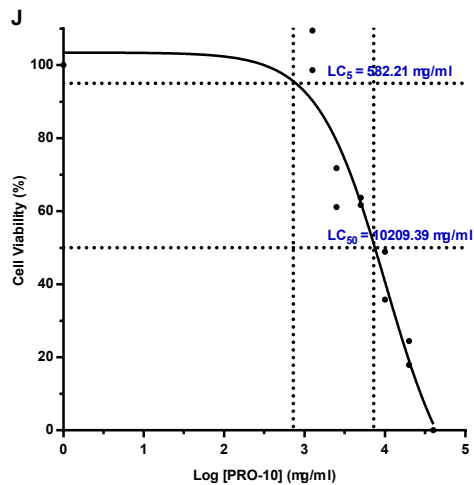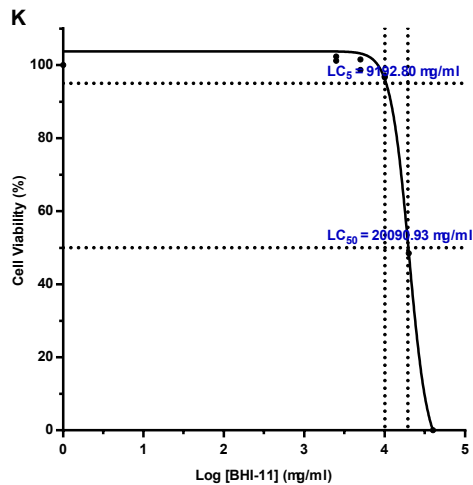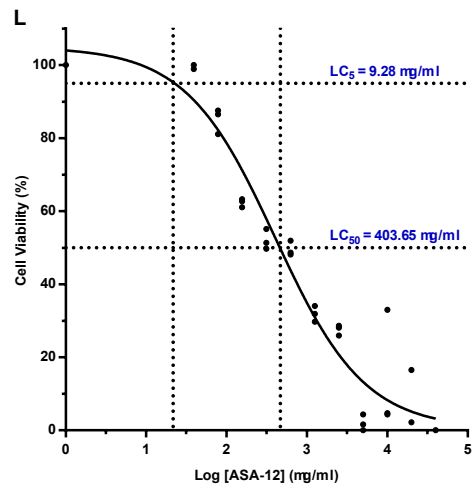

Supplement: Supplemental Information 1 — The 50% lethal concentration, (LC50) and 5% lethal concentration, (LC5) value of 10 LCFS of Lactobacillus isolates on 264.7 cells. [file peerj-09-12586-s001.pdf]
