# Supplementary material for: Lyophilized cell-free supernatants of Lactobacillus isolates exhibited antibiofilm, antioxidant, and reduces nitric oxide activity in lipopolysaccharide-stimulated RAW 264.7 cells
Source: PeerJ. 2021 Nov 30;9:e12586. doi: 10.7717/peerj.12586 (PMC8641486; doi:10.7717/peerj.12586)
Supplement: Supplemental Information 2 [file peerj-09-12586-s002.docx]

**Table S1.** Percentage of the biofilm inhibition of CFS against *A. buamannii* and *E. coli* when compared with the control

| LAB | Percentage of biofilm inhibition (mean±SD) | | | | | |
| --- | --- | --- | --- | --- | --- | --- |
|  | *A. buamannii* | | | *E. coli* | | |
|  | 2 × MIC | 1 × MIC | Control | 2 × MIC | 1 × MIC | Control |
| T0601 | 23.33±3.51^a^ | 15.33±7.64^a^ | 0.00±14.98 | 43.86±1.15 ^a^ | 31.83±9.19 ^a^ | 0.00±3.86 |
| T0602 | 18.67±2.08^a^ | 14.00±5.29^a^ | 0.00±14.98 | 34.09±1.57 ^a^ | 33.58±3.04 ^a^ | 0.00±3.86 |
| T0603 | 3.67±5.51 | 10.00±7.00^a^ | 0.00±14.98 | 32.08±3.13 ^a^ | 33.83±3.28 ^a^ | 0.00±3.86 |
| T0701 | 13.33±2.89^a^ | 11.67±3.79^a^ | 0.00±14.98 | 34.09±3.86 ^a^* | 18.80±5.42 ^a^ | 0.00±3.86 |
| T0802 | 29.33±1.15^a^* | 22.00±1.00^a^ | 0.00±14.98 | 41.35±4.19 ^a^ | 26.32±5.97 ^a^ | 0.00±3.86 |
| T0901 | 14.00±2.00^a^ | 12.67±4.16^a^ | 0.00±14.98 | 30.08±3.45 ^a^ | 30.83±1.30 ^a^ | 0.00±3.86 |
| T0902 | 2.00±7.55 | 5.67±5.69 | 0.00±14.98 | 28.07±1.57 ^a^ | 30.58±1.74 ^a^ | 0.00±3.86 |
| T1301 | 1.00±1.00 | 7.00±2.65 | 0.00±14.98 | 30.33±1.89 ^a^ | 22.56±3.91 ^a^ | 0.00±3.86 |
| T1304 | 26.33±4.73^a^ | 25.33±4.62^a^ | 0.00±14.98 | 31.08±5.44 ^a^* | 14.54±3.78 ^a^ | 0.00±3.86 |
| T1901 | 26.33±1.53^a^ | 21.33±7.23^a^ | 0.00±14.98 | 25.31±1.74 ^a^ | 26.82±0.43 ^a^ | 0.00±3.86 |

a= Significant difference, compared with the negative control

*= Significant difference, compared with the 1 × MIC
