# Supplementary material for: Lyophilized cell-free supernatants of Lactobacillus isolates exhibited antibiofilm, antioxidant, and reduces nitric oxide activity in lipopolysaccharide-stimulated RAW 264.7 cells
Source: PeerJ. 2021 Nov 30;9:e12586. doi: 10.7717/peerj.12586 (PMC8641486; doi:10.7717/peerj.12586)
Supplement: Supplemental Information 3 [file peerj-09-12586-s003.docx]

**Table S2.** Activity of CFS on the eradication of established biofilm of *A. buamannii* and *E. coli* when compared with the control

| LAB | Percentage of the eradication (mean±SD) | | | | | |
| --- | --- | --- | --- | --- | --- | --- |
|  | *A. buamannii* | | | *E. coli* | | |
|  | 2 × MIC | 1 × MIC | Control | 2 × MIC | 1 × MIC | Control |
| T0601 | 40.97±9.16^a^ | 40.05±7.73 ^a^ | 0.00±5.85 | 70.75±4.18 ^a^ | 68.28±6.57 ^a^ | 0.00±6.55 |
| T0602 | 36.76±5.49 ^a^ | 38.60±2.28 ^a^ | 0.00±5.85 | 80.81±3.30 ^a^ | 71.40±9.50 ^a^ | 0.00±6.55 |
| T0603 | 33.20±5.48 ^a^ | 32.54±6.30 ^a^ | 0.00±5.85 | 81.65±5.02 ^a^ | 70.47±11.80 ^a^ | 0.00±6.55 |
| T0701 | 29.64±11.31 ^a^ | 41.63±4.62 ^a^ | 0.00±5.85 | 80.38±5.36 ^a^ | 71.28±3.28 ^a^ | 0.00±6.55 |
| T0802 | 46.77±9.12 ^a^ | 43.87±0.68 ^a^ | 0.00±5.85 | 72.76±7.44 ^a^ | 59.61±7.69 ^a^ | 0.00±6.55 |
| T0901 | 37.29±8.22 ^a^ | 44.27±8.30 ^a^ | 0.00±5.85 | 78.95±0.79 ^a^ | 64.28±8.31 ^a^ | 0.00±6.55 |
| T0902 | 30.30±7.57 ^a^ | 36.89±13.24 ^a^ | 0.00±5.85 | 78.18±7.58 ^a^ | 79.57±6.60 ^a^ | 0.00±6.55 |
| T1301 | 35.97±0.40 ^a^* | 47.43±4.01 ^a^ | 0.00±5.85 | 75.55±3.87 ^a^* | 75.15±3.85 ^a^ | 0.00±6.55 |
| T1304 | 50.99±1.98 ^a^ | 56.52±5.14 ^a^ | 0.00±5.85 | 78.21±2.07 ^a^* | 63.66±3.97 ^a^ | 0.00±6.55 |
| T1901 | 62.98±3.54 ^a^ | 54.94±13.67 ^a^ | 0.00±5.85 | 84.34±0.98 ^a^* | 58.87±7.18 ^a^ | 0.00±6.55 |

a= Significant difference, compared with the negative control

*= Significant difference, compared with the 1 × MIC
